# Inhibition of discoidin domain receptors by imatinib prevented pancreatic fibrosis demonstrated in experimental chronic pancreatitis model

**DOI:** 10.1038/s41598-021-92461-z

**Published:** 2021-06-18

**Authors:** Sapana Bansod, Mohd Aslam Saifi, Chandraiah Godugu

**Affiliations:** 1 Department of Regulatory Toxicology, National Institute of Pharmaceutical Education and Research (NIPER), Balanagar, Hyderabad, Telangana 500037 India; 2 Division of Oncology, Department of Internal Medicine, Washington University School of Medicine, St Louis, MO USA

**Keywords:** Molecular biology, Gastrointestinal diseases, Inflammation, Cytokines

## Abstract

Discoidin domain receptors (DDR1 and DDR2) are the collagen receptors of the family tyrosine kinases, which play significant role in the diseases like inflammation, fibrosis and cancer. Chronic pancreatitis (CP) is a fibro-inflammatory disease in which recurrent pancreatic inflammation leads to pancreatic fibrosis. In the present study, we have investigated the role of DDR1 and DDR2 in CP. The induced expression of DDR1 and DDR2 was observed in primary pancreatic stellate cells (PSCs) and cerulein-induced CP. Subsequently, the protective effects of DDR1/DDR2 inhibitor, imatinib (IMT) were investigated. Pharmacological intervention with IMT effectively downregulated DDR1 and DDR2 expression. Further, IMT treatment reduced pancreatic injury, inflammation, extracellular matrix deposition and PSCs activation along with inhibition of TGF-β1/Smad signaling pathway. Taken together, these results suggest that inhibition of DDR1 and DDR2 controls pancreatic inflammation and fibrosis, which could represent an attractive and promising therapeutic strategy for the treatment of CP.

## Introduction

Chronic pancreatitis (CP) is a fibro-inflammatory disease of the pancreas, characterized by recurrent pancreatic inflammation and fibrosis leading to irreversible impairment of exocrine and endocrine functions of pancreas^1^. CP is a major risk factor for the progression of diabetes and pancreatic cancer in the developed countries^2^. Although there has been an extensive research in CP during last few years, but the exact pathogenesis and specific molecular mechanism of CP needs to be elucidated. The well-established theory behind CP development is premature activation of pancreatic digestive enzymes leading to autodigestion of pancreatic tissue and the recruitment of the excess inflammatory cells that results in pancreatic inflammation^3^. Pancreatic fibrosis is a complex pathological event in which wherein dysregulation of production and degradation of extracellular matrix (ECM) is observed in the pancreas. Persistent inflammation causes the activation of pancreatic fibroblasts, known as pancreatic stellate cells (PSCs) which promote development of pancreatic fibrosis. Upon activation, PSCs undergo morphological changes and initiate pancreatic fibro-inflammation through the secretion of a number of inflammatory mediators and excessive synthesis of ECM proteins such as collagen I, III and fibronectin^4^. Among these mediators, transforming growth factor (TGF)-β1 is the potent profibrotic cytokine which regulates PSCs activation and pancreatic fibrogenesis through its downstream Smad signaling pathway^5^. Currently, CP treatment is only limited to the supportive care and there is no clinically available therapeutic agent to treat CP and associated fibrosis progression. Thus, there is a pressing need to identify novel therapeutic agents for the treatment of CP on high priority, which could be helpful to reduce costs of long-term therapy and improve the quality of life.

Discoidin domain receptors (DDR1 and DDR2) are new class of tyrosine kinase receptors, which get activated in response to collagens and are involved in the early embryonic development^6^. DDR1 is majorly expressed in epithelial cells and activated by both fibrillar and nonfibrillar collagens (type I to V, VIII, and XI), while DDR2 is expressed in fibroblasts and activated by fibrillar collagens (type I and III). This binding causes phosphorylation and dimerization of the tyrosine kinase receptors which then leads to the activation of different signaling pathways involved in inflammation and fibrosis^7^. Accumulating evidences have reported that upregulated expression of DDR1 and DDR2 are observed in the chronic injury, inflammation and fibrotic conditions^8^. Multiple studies reported that inhibition of DDR1 reduced deposition of collagen I and IV in alcoholic liver fibrosis, lung fibrosis, cirrhotic liver and chronic nephropathies^9–11^. On the other hand, DDR2 is a key regulator of epithelial to mesenchymal transition (EMT) program, which is involved in several pathological changes such as fibrosis and tumor progression^12^. Although, the DDRs are involved in inflammatory and fibrotic diseases but their role in CP and associated fibrosis is not explored fully.

Imatinib mesylate (IMT) is a standard Food and Drug Administration (FDA)-approved drug for the treatment of patients with chronic myelocytic leukemia. IMT is the only tyrosine kinase inhibitor which potentially inhibits both the profibrotic cytokines, TGF-β and PDGF which are involved in regulation of organ fibrosis process. Emerging studies have shown that IMT has the potent tyrosine kinase DDR1 and DDR2 inhibitory activity^13^. IMT is the type-2 DDRs inhibitor which inhibits tyrosine kinase domain by leveraging the ATP binding site as well as the allosteric site, which is freely accessible in the inactive conformation^14^. However, the effect of IMT on CP has not been studied yet and the underlying molecular mechanism remains to be explored. Therefore, in the present study, we investigated the role of DDR1 and DDR2 in progression of CP and associated fibrosis. In addition, we investigated the protective effects of pharmacological inhibitor of DDR1 and DDR2, IMT against CP and pancreatic fibrosis. Our results showed that inhibition of DDR1 and DDR2 signaling by IMT could effectively attenuate CP and associated fibrosis and further indicate that DDR1 and DDR2 could be the potential therapeutic targets for treatment of CP.

## Materials and methods

### Reagents

Imatinib mesylate (IMT) was procured from Sigma Aldrich, USA. Cerulein was obtained from Ana Spec, France. TGF-β1 was procured from Bio-legend, USA. The antibodies viz. β-actin (Catalogue no. sc-47778), Collagen1a (Catalogue no. sc-393573), Collagen3a (Catalogue no. sc-271249), α-SMA (Catalogue no. sc-53142), CTGF (Catalogue no. sc-373936), TGF-β1 (Catalogue no. sc-146), DDR2 (Catalogue no. sc-81707), pSmad2/3 (Catalogue no. #8828), Smad2/3 (Catalogue no. #8685), pDDR1 (Catalogue no. #14531), DDR1 (Catalogue no. #5583), pNF-κB (Catalogue no. #3033), NF-κB (Catalogue no. #8242) were procured from Santa Cruz Biotechnology Ltd and Cell Signaling Technology, USA. The ELISA kits of TGF-β, IL-1β, IL-6 and TNF-α (Catalogue no. TGF-β: 88–8350, IL-1β: 88–7013, IL-6: 88- 7064, and TNF-α: 88–7324) were obtained from eBioscience, USA.

### PSCs isolation, culture and treatment

PSCs were isolated from the mice according to the methods as described with slight modifications^15^. Briefly, pancreas was isolated from adult male Swiss albino mice. Isolated pancreas was immediately washed in sterile PBS. A small portion of pancreas was cut, minced with scissors and single cell suspension was prepared using 1 ml syringe in Dulbecco’s Modified Eagle medium (DMEM). Next, cell suspension was centrifuged followed by two times washing with PBS. Cell’s pellet was mixed and cultured in DMEM medium containing 1% antibiotic solution and supplemented with 20% fetal bovine serum (FBS). PSCs were separated by using the Histodenz density gradient centrifugation method^16^.

### Oil-red O staining for PSCs identification

After 70% confluency, PSCs were fixed with 4% paraformaldehyde and 0.1% Triton X-100 reagents and stained with oil-red O working solution (1% in isopropanol) at 60 ℃ for 30 min. Next, PSCs were counterstained using hematoxylin, mounted with mounting medium, Dibutylphthalate Polystyrene Xylene (DPX) and observed under the microscope^17^.

### Confocal microscopy

Freshly isolated and cultured PSCs were treated with IMT (1 μM) in the presence or absence of TGF-β1 (10 ng/ml). After 24 h of treatment, cells were fixed with 4% paraformaldehyde and 0.1% Triton X-100 followed by blocking with 3% bovine serum albumin (BSA) for 1 h. The cells were then labeled with primary antibodies against α-SMA, collagen 1a, pSmad2/3, pDDR1, and DDR2 at 4 °C for overnight. Next day, cells were probed with secondary antibody conjugated with rhodamine (1:200 dilution) or fluorescein isothiocyanate (FITC) (1:100 dilution) for 1 h. After washing, cells were mounted with fluoroshield 4′,6-diamidino-2-phenylindole (DAPI) (Sigma-Aldrich, USA) medium and fluorescent signals were captured by confocal laser-scanning microscopy (Leica, Germany)^18^.

### Animals and experimental design

Animals were kept in well-controlled housing facility at temperature (25 ± 2 °C) and 12/12-h light/dark cycle with free access to water and pellet food. The Animal experiments were designed, conducted and reported as per the ARRIVE guidelines^19^. Specifically, all the animal experiments were performed according to the Committee for the Purpose of Control and Supervision of Experiments on Animals (CPCSEA), which is the approval body for animal experimentation in India. The CPCSEA certified Institutional Animal Ethical Committee (IAEC) of National Institute of Pharmaceutical Education and Research (NIPER)-Hyderabad, has reviewed the animal protocol and approved it (IAEC Protocol Approval No.: NIP/01/2019/RT/365).

**Figure 1:**
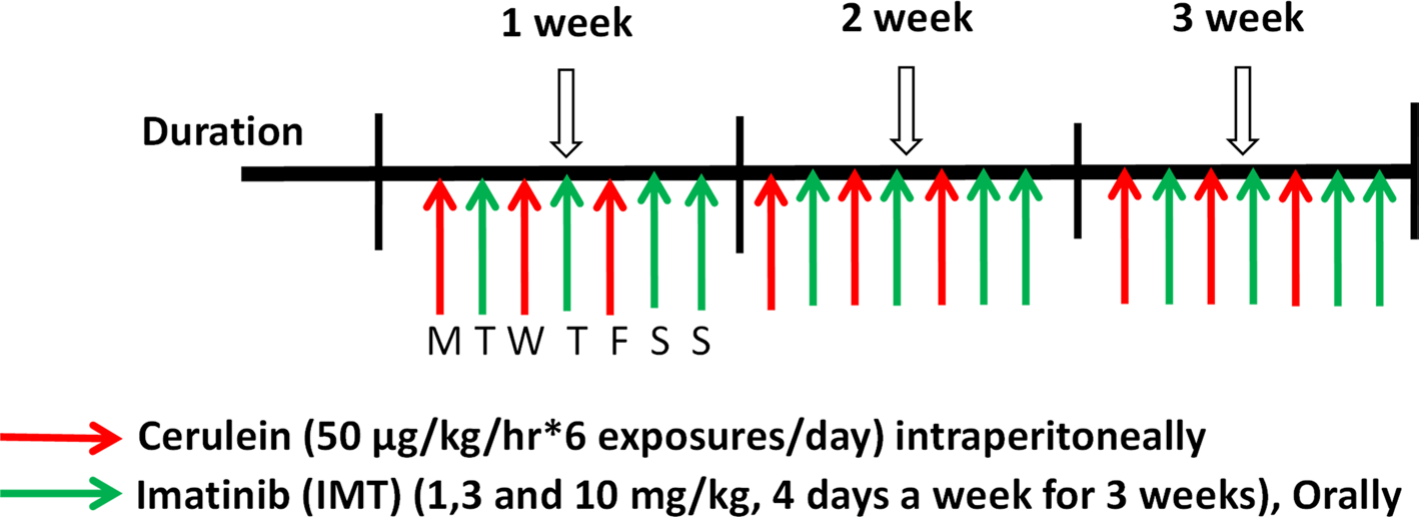
Experimental design for development and treatment of CP in mice. Male Swiss albino mice were randomly divided into 6 groups (7 mice per group): Normal control, IMT alone, Cerulein, Cerulein + IMT low dose (IMT LD—1 mg/kg), Cerulein + IMT mid dose (IMT MD—3 mg/kg) and Cerulein + IMT high dose (IMT HD—10 mg/kg). CP was induced by 6-hourly intraperitoneal (i.p.) injections of cerulein (50 μg/kg), 3 alternative days per week, for the period of 3 weeks. Cerulein + IMT group mice were administered IMT (1, 3 and 10 mg/kg) orally, every other day after cerulein exposures for 3 weeks. Normal control group mice received normal saline same as cerulein group. IMT alone group mice were administrated with IMT (10 mg/kg) orally 4 alterative days in the week for 3 weeks.

Male Swiss albino mice (age: 6–8 weeks, body weight 25–30 g) were purchased from Palamur Biosciences Pvt. Ltd, Mahabubnagar, India. All mice were randomly divided into 6 groups (7 mice per group, n = 42): Normal control, IMT alone, Cerulein, Cerulein + IMT low dose (LD—1 mg/kg), Cerulein + IMT mid dose (MD—3 mg/kg) and Cerulein + IMT high dose (HD—10 mg/kg). CP was induced by 6-hourly exposures of cerulein (50 μg/kg/intraperitoneal (i.p.), 3 alternative days per week, for the period of 3 weeks as per our previously published protocol^20^. Cerulein + IMT group mice were administered IMT (1, 3 and 10 mg/kg) orally, every other day after cerulein exposures for 3 weeks. Normal control animals received i.p. injection of sterile normal saline same as cerulein group. IMT alone group mice were administrated with IMT (10 mg/kg) orally 4 alterative days in the week for 3 weeks (Fig. 1). Mice were sacrificed via inhalation euthanasia (CO_2_ asphyxiation) after completion of 21 days. Doses of IMT were selected on the basis of previous literature^21^. IMT was dissolved in distilled water and administrated orally 4 alterative days in the week for 3 weeks. As cerulein needed minimum 12 h to produce its maximum inflammatory response, IMT was given every other day after cerulein exposures for a period of 3 weeks^20^.

### Blood collection and plasma analysis

Blood samples were collected by cardiac puncture on the day of sacrifice in heparin containing tubes and plasma was separated after centrifugation. Amylase and lipase levels were assessed in plasma and measured by using Accurex kinetic enzymatic kits and values are expressed as IU/L^20,22^.

### Collagen estimation by Sircol assay

Pancreatic tissues were homogenized in PBS and then supernatants were incubated with collagen binding dye picrosirius red for 1 h at 37 °C. Next, samples were centrifuged and obtained pellet was resuspended in 100% ethanol followed by centrifugation at 9168×*g* for 10 min. Then pellet was mixed with 0.5 M NaOH solution and incubated for 30 min at 37 °C. The final absorbance was taken at 540 nm and values were expressed as relative collagen content per milligram of protein^23^.

### Enzyme-linked immunosorbent assay

Cytokine concentrations in pancreas were determined by eBioscience ELISA kits for IL-6, IL-1β, TNF-α and TGF-β1 as per the previously described protocol^24^. Concentration of cytokines was expressed as pg per milligram of protein. Total protein was estimated by Bicinchoninic acid assay kit (Sigma Aldrich, USA).

### Histopathology and immunohistochemistry analysis

Pancreas were fixed in 10% formaldehyde solution and paraffin-embedded pancreatic sections were taken at 5 µm using microtome (Leica, Germany). The pancreatic sections were stained with H & E, picrosirius red (PSR) and Masson’s trichrome (MT) as per previously described protocol^25^. For immunostaining, antigen retrieval was carried out by citrate buffer. After washing, sections were incubated with 3% hydrogen peroxide for 15 min followed by blocking with 3% BSA. The primary antibodies against α-SMA, collagen1a, pDDR1 and DDR2 (1:100) were added and kept at 4 ℃ overnight. The HRP-conjugated secondary antibodies were added and sections were stained with 3, 3-diaminobenzidine (DAB) reagent. Further, sections were counterstained with hematoxylin and images were observed using a light microscope (CX21i Olympus, India)^26^. Quantitative analysis of fibrotic and immunopositive area were analyzed using ImageJ software (NIH, USA).

### Immunoblotting

To analyze the pancreas protein expression, immunoblotting technique was used as described earlier^27,28^. Small portions of pancreatic tissues were homogenized in RIPA lysis buffer. Then equal amounts of proteins were separated using SDS-PAGE gel electrophoresis and transferred to a nitrocellulose membrane. The protein blots were then detected using ECL (Bio-Rad Laboratories, USA) and densitometric analysis of respective protein bands was carried out by ImageJ software (NIH, USA).

### Statistical analysis

All results are given as mean ± SEM. Student’s t-test was used to determine the difference between two groups. However, differences among more than two groups were analyzed using nonparametric test one-way analysis of variance by GraphPad Prism scientific software version 6.01. Value of P less than 0.05 was considered statistically significant.

## Results

### Upregulation of DDR1 and DDR2 expression in pancreatic fibrosis

**Figure 2:**
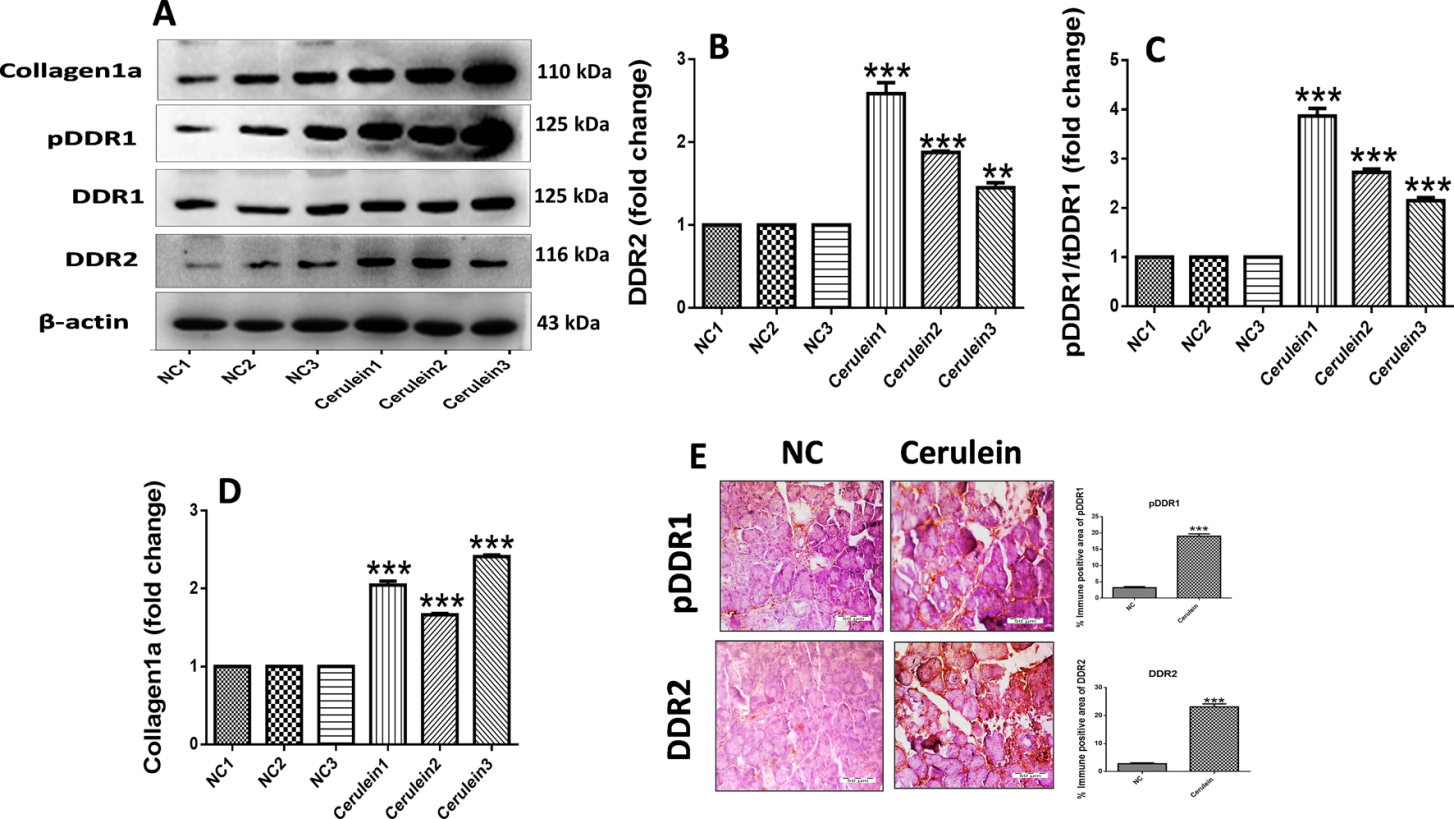
Upregulation of DDR1 and DDR2 in pancreatic fibrosis. (**A–D**) Western blot and corresponding densitometric analysis of protein expression of phosphorylated DDR1, total DDR1, DDR2 and collagen1a in pancreatic tissue. (**E**) Representative photographs of immunohistochemistry of phosphorylated DDR1 and DDR2 in the pancreatic tissue. β-actin was used as loading control. All values are represented as mean ± SEM (n = 3–10). Statistical significance was established using Student’s t-test where **P < 0.01, ***P < 0.001 vs Normal control. Full-length blots are presented in Supplementary Fig. S1.

We evaluated the expression of DDR1 and DDR2 via western blot and immunohistochemical analysis in CP mice. Interestingly, we found marked upregulation of phosphorylated DDR1, DDR2 and collagen1a expression in pancreatic tissue of CP mice (Fig. 2A–D, Supplemental Fig. S1). In addition, immunohistochemical (IHC) staining and quantification analysis demonstrated that expression of DDR1 and DDR2 was significantly increased in CP model mice as compared to the normal control mice (Fig. 2E). These results indicated the upregulation of DDR1 and DDR2 receptors in the progression of CP and associated pancreatic fibrosis.

### Inhibition of DDR1 and DDR2 prevents progression of pancreatic fibrosis

**Figure 3:**
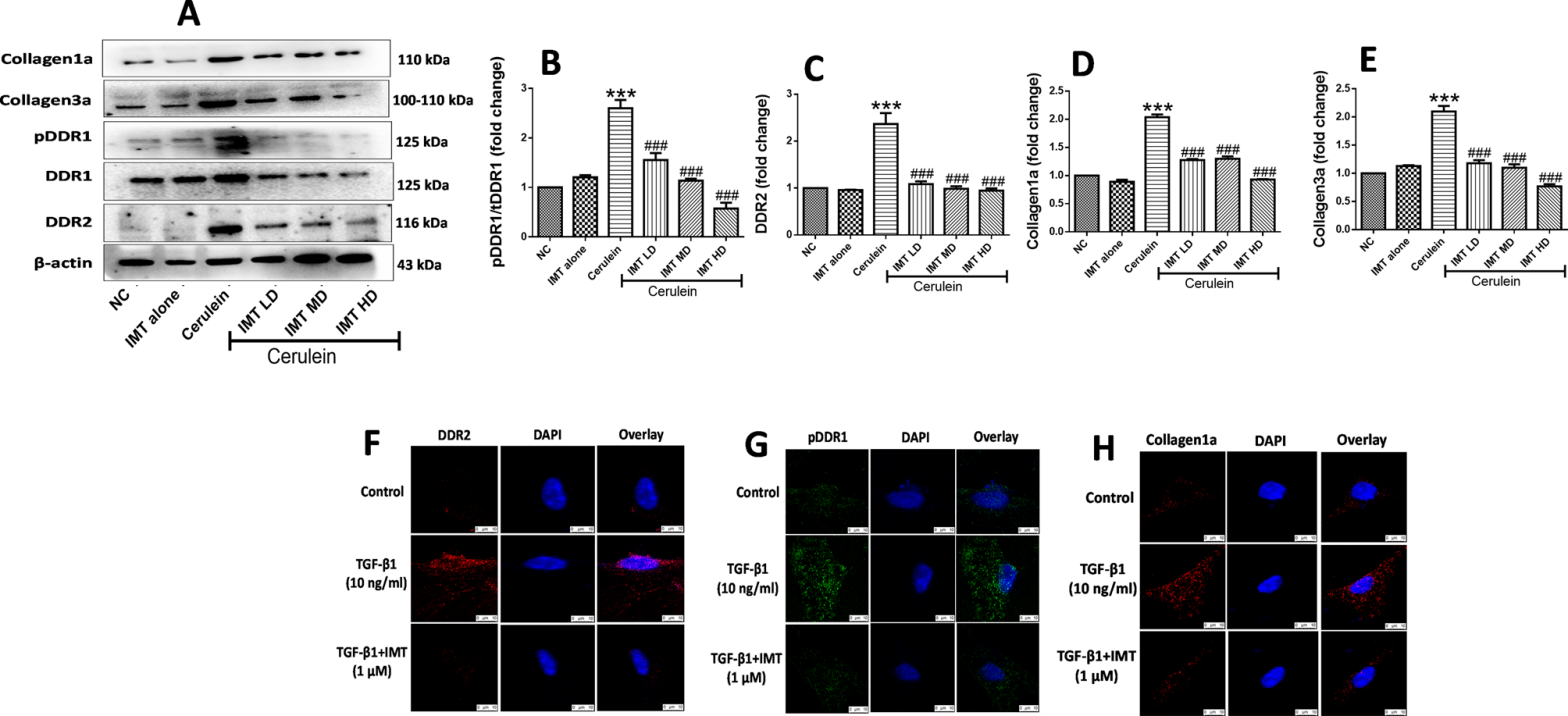
Inhibition of DDR1 and DDR2 expression prevents progression of pancreatic fibrosis. (**A–E**) Western blot and corresponding densitometric analysis of protein expression of phosphorylated DDR1, total DDR1, DDR2, collagen1a and collagen3a in pancreatic tissue. (**F–H**) Immunofluorescence (IF) staining for phosphorylated DDR1, DDR2 and collagen1a in TGF-β1-induced PSCs. β-actin was used as loading control. All values are represented as mean ± SEM (n = 3). Statistical significance was established using one-way ANOVA followed by Tukey’s multiple comparisons test where ***P < 0.001 vs Normal control, ^###^P < 0.001 vs Cerulein. Full-length blots are presented in Supplementary Fig. S2.

Next, to investigate whether collagen receptors DDR1 and DDR2 play key role in the development of CP and associated fibrosis or not, we evaluated the effects of DDR1/DDR2 inhibitor, IMT in cerulein-induced CP model. Treatment with IMT (1, 3 and 10 mg/kg) showed significant downregulation of phosphorylated DDR1 and DDR2 expression in a dose-dependent manner (Fig. 3A–C, Supplemental Fig. S2). In addition, we found that cerulein-treated mice demonstrated significantly higher collagen expression, whereas oral administration of IMT dose-dependently reduced collagen expression as compared to cerulein-control mice (Fig. 3D,E). We further assessed the effects of IMT on TGF-β1-induced PSCs activation by confocal microscopy. Our results demonstrated that TGF-β1-treated PSCs showed notably upregulated pDDR1, DDR2 and collagen1a expression and the expression of these proteins was markedly inhibited by IMT treatment (Fig. 3F,G). Together, these results suggest that inhibition of DDR1 and DDR2 expression suppressed collagen deposition and pancreatic fibrosis.

### IMT controls collagen deposition in the pancreas

**Figure 4:**
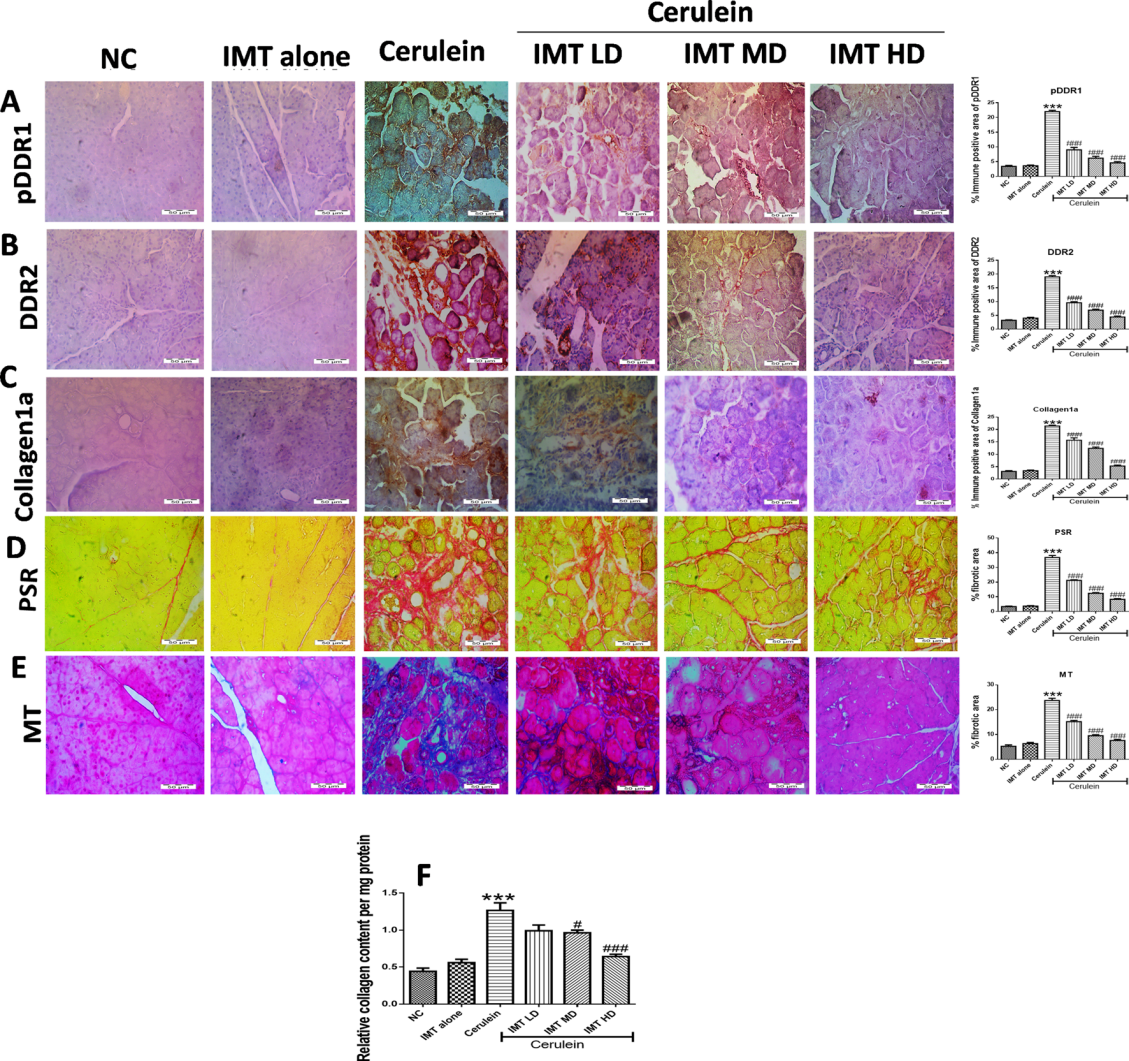
IMT controls collagen deposition in the pancreas (**A–C**) Representative photographs and quantitative analysis of immunohistochemistry of phosphorylated DDR1, DDR2 and collagen1a protein, (**D,E**) Representative photographs and quantitative analysis of PSR and MT staining in the pancreatic tissue, (**F**) Relative collagen content in pancreas estimated by Sircol assay. All values are represented as mean ± SEM (n = 10). Statistical significance was established using one-way ANOVA followed by Tukey’s multiple comparisons test where ***P < 0.001 vs Normal control, ^#^P < 0.05, ^###^P < 0.001 vs Cerulein.

IHC staining and their quantification analysis showed that the expression of DDR1, DDR2 and collagen1a were strongly increased in CP mice. However, IMT treatment prevented the elevated pancreatic DDR1, DDR2, and collagen1a expression as confirmed by IHC analysis (Fig. 4A–C). Further, pancreatic sections of cerulein-treated mice showed marked pancreatic fibrosis illustrated by red and blue color in PSR and MT staining, respectively around the pancreatic ducts. Interestingly, treatment with DDRs inhibitor, IMT significantly attenuated collagen deposition in the pancreas (Fig. 4D,E). In addition, Sircol assay was performed to measure total collagen content in the pancreas. Our results demonstrated that CP mice demonstrated significantly increased collagen content and this collagen content was effectively reduced by IMT treatment (Fig. 4F).

### IMT attenuates cerulein-induced pancreatic injury and inflammation

**Figure 5:**
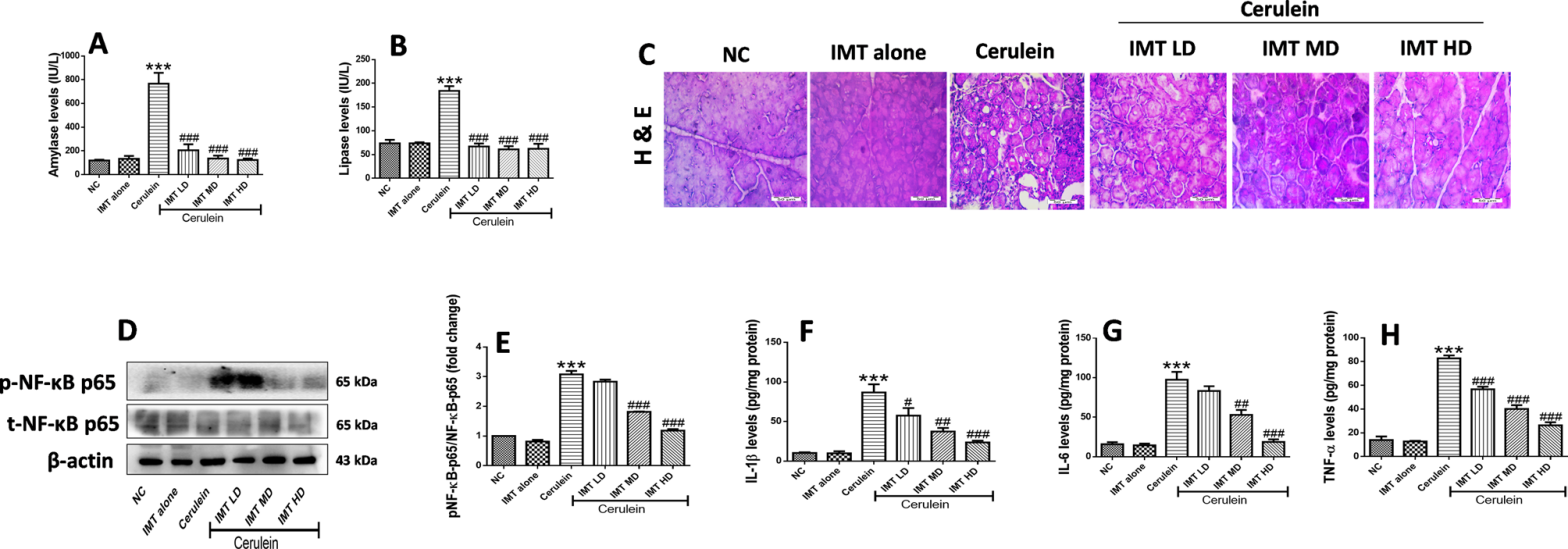
Effect of IMT on cerulein-induced pancreatic injury and inflammation. (**A,B**) Plasma amylase and lipase levels. (**C**) Representative photographs of H & E staining of pancreas at 400X magnification. (**D,E**) Western blot and corresponding densitometric analysis of protein expression of total and phosphorylated NF-κB in pancreas, (**F–H**) Pancreatic IL-1β, IL-6 and TNF-α levels studied by ELSA. β-actin was used as loading control. All values are represented as mean ± SEM (n = 7). Statistical significance was established using one-way ANOVA followed by Tukey’s multiple comparisons test where ***P < 0.001 vs Normal control, #P < 0.05, ^##^P < 0.01, ^###^P < 0.001 vs Cerulein. Full-length blots are presented in Supplementary Fig. S3.

Next, we investigated the protective effects of IMT against cerulein-induced CP model. Cerulein challenged mice demonstrated significant elevation in plasma amylase and lipase levels, while IMT administration effectively reduced these levels (Fig. 5A,B). Further, to evaluate the anti-inflammatory effects of IMT, histopathological examination was performed in the pancreatic tissue by H&E staining. Our results revealed that the hallmark characteristics of CP including acinar cell atrophy, vacuolization, inflammatory cells infiltration and collagen deposition were observed in the cerulein control pancreas. On the other hand, pharmacological intervention with IMT markedly ameliorated cerulein-induced histopathological alterations as shown in Fig. 5C. These results suggest that IMT attenuated cerulein-induced pancreatic injury in CP mice. Furthermore, we examined the effect of IMT on NF-κB p65 expression by western blot analysis. We found that the expression of phosphorylated NF-κB p65 was significantly increased in cerulein challenged mice, while IMT mid dose and high dose mice demonstrated significant inhibition of phosphorylated NF-κB expression in the pancreatic tissues (Fig. 5D,E, Supplemental Fig. S3). In addition to this, we observed that repetitive cerulein exposure significantly increased the cytokine production in the pancreas, while IMT treatment markedly suppressed these cytokines levels, indicating downregulation of local production of pro-inflammatory cytokines (Fig. 5F–H). Collectively, our results indicated that IMT shows potent anti-inflammatory effects and prevent pancreatic injury in CP mice.

### IMT inhibits the activation of PSCs studied in-vivo and in-vitro model

**Figure 6:**
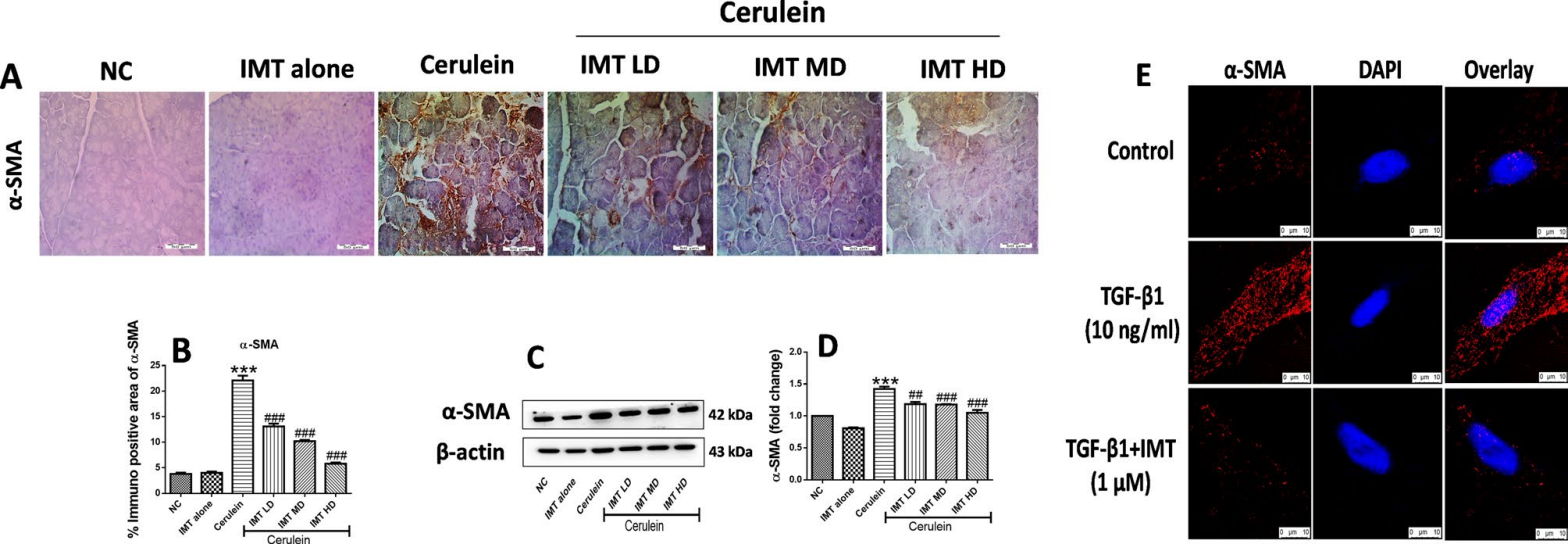
Effect of IMT on pancreatic stellate cells activation (PSCs). (**A,B**) Representative photographs of immunohistochemistry and quantitative analysis of α-SMA protein in the pancreatic tissue, (**C,D**) Western blot and corresponding densitometric analysis of protein expression of α-SMA, (**E**) Immunofluorescence (IF) staining for α-SMA in TGF-β1-induced PSCs. β-actin was used as loading control. All values are represented as mean ± SEM (n = 3–10). Statistical significance was established using one-way ANOVA followed by Tukey’s multiple comparisons test where ***P < 0.001 vs Normal control, ^##^P < 0.01, ^###^P < 0.001 vs Cerulein. Full-length blots are presented in Supplementary Fig. S4.

PSCs activation is a major event involved in the synthesis and accumulation of ECM proteins resulting in the development of pancreatic fibrosis. In response to chronic injury, quiescent PSCs convert to an activated PSCs synthesizing excessive ECM proteins. Our in-vivo results showed that the expression of α-SMA was profoundly increased in cerulein-treated CP mice, indicating increased number of activated PSCs during CP. On the other hand, pharmacological treatment with IMT significantly inhibited the PSCs activation as demonstrated by downregulation of α-SMA expression in the pancreas (Fig. 6A–D, Supplemental Fig. S4). Consistent with the in-vivo results, we observed marked upregulation of α-SMA expression in TGF-β1-stimulated mouse PSCs. However, treatment with IMT successfully decreased α-SMA expression, indicating the effective inhibition of PSCs activation by IMT (Fig. 6E). These results clearly indicated that IMT successfully inhibited PSCs activation.

### IMT inhibits TGF-β/Smad signaling

**Figure 7:**
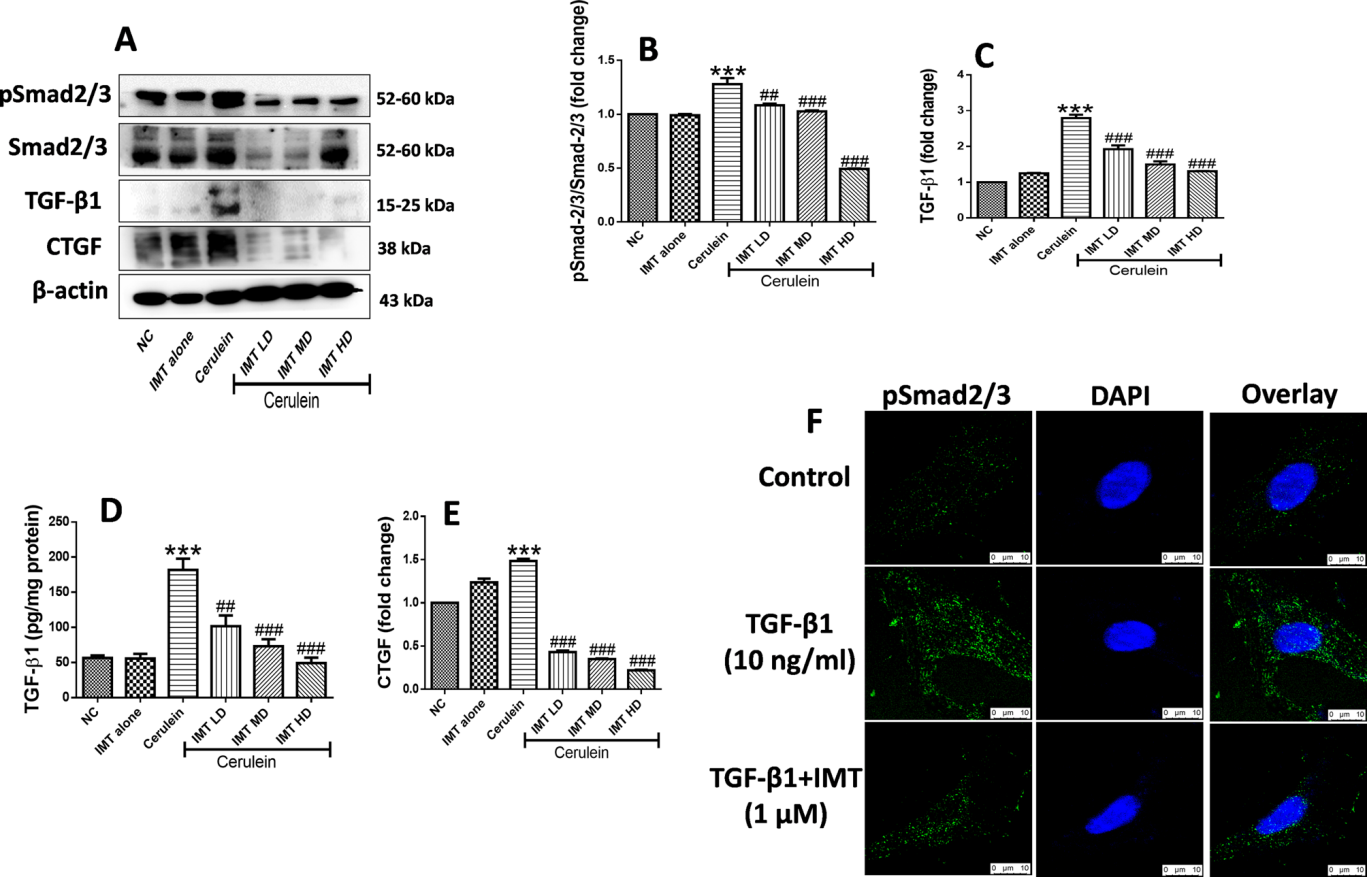
Effect of IMT on TGF-β1/Smad signaling pathway. (**A**–**D**) Western blot and corresponding densitometric analysis of protein expression of phosphorylated and total Smad2/3, TGF-β1 and CTGF in pancreatic tissue, (**E**) Pancreatic TGF-β1 levels studied by ELISA. (**F**) IF staining for phosphorylated Smad2/3 in TGF-β1-induced PSCs. β-actin was used as loading control. All values are represented as mean ± SEM (n = 3). Statistical significance was established using one-way ANOVA followed by Tukey’s multiple comparisons test where ***P < 0.001 vs Normal control, ^##^P < 0.01, ^###^P < 0.001 vs Cerulein. Full-length blots are presented in Supplementary Fig. S5.

TGF-β/Smad is a well-established fibrotic signaling pathway which gets activated in response to the injuries (Fig. 7A, Supplemental Fig. S5). Further, we found that IMT treatment significantly downregulated the expression of phosphorylated Smad2/3 in CP mice (Fig. 7B). In addition to this, our results demonstrated that there was a significant increase in TGF-β1 expression in cerulein- challenged mice. Treatment with IMT significantly inhibited expression of TGF-β1 in a dose-dependent manner (Fig. 7C,D). Furthermore, our western blot results revealed the expression of CTGF was markedly increased in cerulein-induced CP mice, which was effectively inhibited by IMT treatment (Fig. 7E). On the other hand, TGF-β1-induced PSCs also showed marked increase in phosphorylated Smad2/3 expression and this expression was effectively downregulated by IMT treatment (Fig. 7F). Taken together, these results indicated that IMT significantly inhibited TGF-β/Smad signaling in CP model and in PSCs.

## Discussion

CP is a fibro-inflammatory disease which is majorly associated with abnormal synthesis and deposition of ECM in the pancreas leading to the development of pancreatic fibrosis^18,20,25^. Although, there are multiple signaling pathways involved in the pathogenesis of CP, activation of collagen-producing PSCs is the foremost event in the progression of pancreatic fibrosis. PSCs are the main cells of pancreas involved in the ECM deposition and activation of fibrotic signaling during pancreatic fibrosis. It is well-known that TGF-β1, a profibrotic cytokine induces the activation of PSCs resulting in excess synthesis and secretion of collagen by PSCs consequently leading to pancreatic fibrosis^29^.

Collagen-activated receptors, DDRs gained a considerable amount of attention because of their involvement in the multiple pathological conditions and are classified into 2 types: DDR1 and DDR2^30–32^. These receptors are majorly involved in both physiological as well as pathological processes^33^. Recently, studies have reported that collagen receptors DDR1 and DDR2 play a central role in the regulation of inflammation and fibrotic conditions^34,35^. Apart from this, DDR1 and DDR2 receptors also play a decisive role in the activation of myofibroblasts and TGF-β/Smad signaling which is majorly involved in the initiation and progression of fibrosis^36^. The hallmark feature of DDRs is that they are directly activated upon binding to components of ECM proteins which then activates inflammatory and fibrotic signaling pathways^37^. Notwithstanding, the exact involvement of DDR1 and DDR2 in CP and associated pancreatic fibrosis remains unclear and has not been studied yet. In CP, repetitive injury of the pancreas trigger release of variety of cytokines and chemokines, which results in the activation of collagen-producing PSCs accompanied by excessive accumulation of ECM.

The significant upregulation of DDRs was observed in the present study which suggested that these receptors play a major role in the development of pancreatic fibrosis. The results were consistent with previous literature where activation of DDR1 and DDR2 was found to be involved in the progression of fibrosis in kidney and lungs^8,34^. In a recent study, Ruggeri et al. reported that overexpression of DDR1 plays a significant role during pancreatic injury, tumor development, and tumor progression^38^. Based on our observations of increased expression of DDR1 and DDR2 in CP conditions, we have selected IMT as a potential inhibitor of these receptors on the basis of its well proven inhibitory effects on these two receptors^14^. Moreover, being clinically available FDA approved drug, it may be relatively easy to develop this drug as possible therapy against CP. The significant inhibition of DDR1 and DDR2 by IMT observed in the present study suggested a strong link between DDRs and pancreatic fibrosis. Our results were found to be in correlation with previous studies showing role of DDRs in fibrosis, e.g., Moll et al., reported that pharmacological inhibition of DDR1 significantly inhibited fibrotic and inflammatory protein expression in the human crescentic glomerulonephritis^39^. Another study demonstrated the reduction in the deposition of collagen in the kidneys of DDR1 null mice. In addition, Li et al. demonstrated that deletion of DDR2 significantly alleviated renal interstitial fibrosis induced by unilateral ureteral obstruction^40^. In light of the results of present as well as previous studies, it was evident that DDRs play a major role in the progression of pancreatic fibrosis and the inhibition of these receptors could become a novel approach for the fibrotic disorders.

The inflammation is one of the most important factors involved in the initiation and progression of the fibrosis in different organs. In context of pancreatic injury, the inflammatory cells infiltration along with acinar cell atrophy are major players in the CP^41^. Although, the DDRs are essential for normal development and tissue homeostasis; the overexpression of these receptors is associated with tissue injury^42^. Similarly, we also observed marked pancreatic injury and histopathological alterations including atrophy of acinar cells, vacuolization, infiltration of inflammatory cells along with induced expression of DDRs. However, the inhibition of DDR1 and DDR2 resulted in reduced pancreatic injury and histopathological alterations in the present study. Our results were consistent with the earlier reports where DDRs were reported to be involved in the regulation of tissue homeostasis, cell proliferation, migration and remodeling of the ECM in the injured pancreas^38^. In addition, we found that CP mice showed activated NF-κB signaling accompanied with elevation of inflammatory mediators namely, TNF-α, IL-1β and IL-6 in the pancreas. There is a high possibility that this increased inflammatory process could be due to the elevated DDR1 and DDR2 expression as the previous reports showed that DDR1 and DDR2 activation was accompanied by the induction of pro-inflammatory cytokines^42,43^. It is highly likely that DDR1 and DDR2 could specifically initiate the release of several inflammatory mediators by the activation of NF-κB in CP pancreas. On the other hand, the inhibition of DDR1 and DDR2 showed anti-inflammatory potential via successful inhibition of the inflammatory signaling in CP mice further supporting the association of DDRs with inflammation^35,44^.

PSCs are the major effector cells which play a central role in the progression and development of pancreatic fibrosis in response to persistent injury^45^. PSCs activate and proliferate in response to profibrotic cytokines such as TGF-β1, CTGF and PDGF^46^. Activated PSCs show induced expression of α-SMA and increases the accumulation of ECM proteins including collagens type I and III, and fibronectin^47^. In fact, the activation of PSCs has been observed in pancreatic injury of humans as well as animals. Haber et al., demonstrated that PSCs are activated in the experimental and human pancreatic fibrosis^48^. In the light of previous study, we observed activation of PSCs in CP mice which could be due to the upregulation of DDR1 and DDR2 proteins. Growing evidences showed that excessive deposition of collagen type I and III activates DDR1 and DDR2 signaling which then induces proliferation and activation of myofibroblasts^49,50^. Similarly, we found that pharmacological inhibition of DDR1 and DDR2 significantly blunted PSCs activation and ECM deposition. Our results were complying with the earlier literature, where inhibition of DDR1 and DDR2 prevented the fibroblast proliferation and migration^30,51^. These results suggest that the suppression of PSCs activation and ECM markers might be the result of the downregulated DDRs’ expression, which ultimately resulted in the attenuation of pancreatic fibrosis. Our findings imply that inhibition of DDR1 and DDR2 could be an attractive molecular target for the prevention of PSCs activation and pancreatic fibrosis.

**Figure 8:**
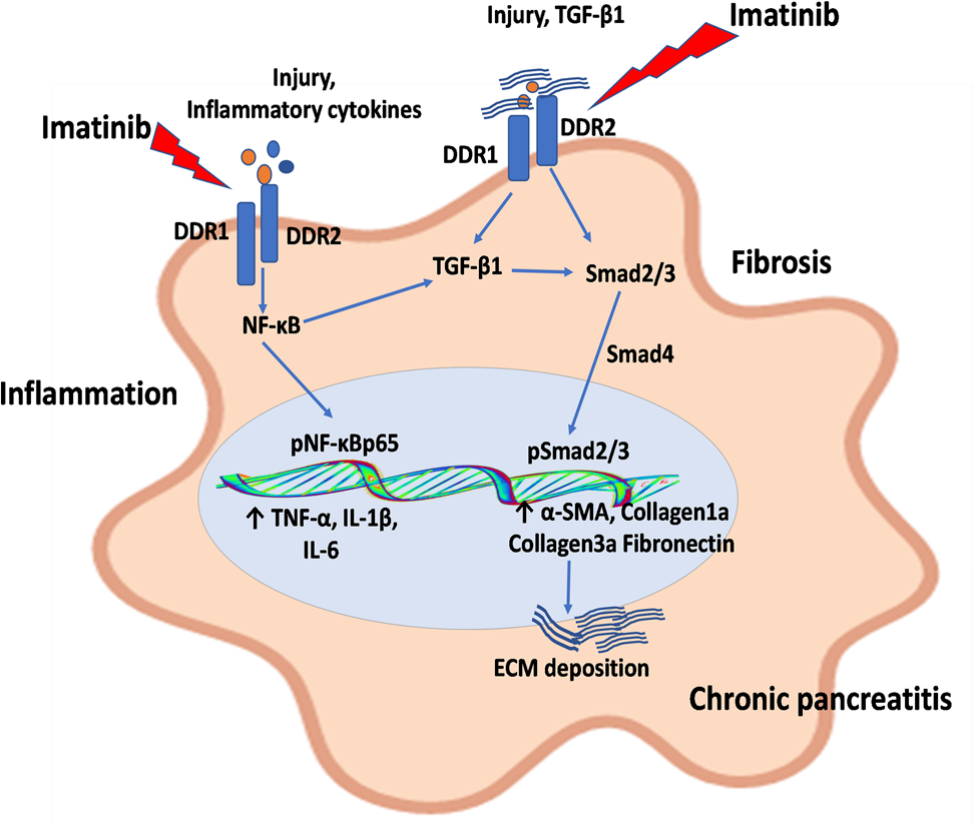
Schematic diagram of the molecular mechanisms of discoidin domain receptors (DDR1/DDR2) in the activation of pancreatic stellate cells (PSCs) and progression of pancreatic fibrosis. During pancreatic injury, release of variety of inflammatory mediators and transforming growth factor (TGF)-β promotes the upregulation of expression of DDR1 and DDR2, which results in the activation of TGF-β1 downstream pathway and NF-κB signaling, consequently leading to excessive ECM deposition and pancreatic fibrosis. Moreover, IMT produced its protective effects by inhibiting DDR1/DDR2 signaling pathways in CP and associated fibrosis.

Growing number of evidences report that TGF-β1 plays a phenomenal role in all types of fibrotic diseases including pancreatic fibrosis. It is the main mediator for the activation of PSCs which subsequently increases deposition of ECM proteins^29^. TGF-β1 binds to its receptors expressed by PSCs and initiates its fibrotic responses through the activation of Smad pathway. In addition, our earlier studies reported that activation of TGF-β/Smad stimulates PSCs activation and consequently results in the progression of the pancreatic fibrosis^18,25^. In context with CP, researchers have documented that there is an activation of TGF-β1/Smad signaling in response to the overexpression of DDR1/DDR2^52^, but its role in the regulation of TGF-β1/Smad signaling and PSCs activation in CP has not been explored. In accordance with the earlier reports, we observed the activation of TGF-β1 and its downstream mediators, which might be due to the upregulation of DDR1 and DDR2 expression in CP. Our results are in accordance with the previous study showing that DDR1 interacts with TGF-β1 pathway to restrict calcifying extracellular vesicle-mediated mineralization and fibrosis in vascular smooth muscle cells^53^. Further, studies have also demonstrated that genetic deletion of DDR1 is directly associated with the downregulation of TGF-β and CTGF in renal fibrosis^43,54^. In addition, Zhao et al., reported that upregulation of DDR2 activates the TGF-β signaling, while DDR2 knockdown resulted in the inhibition of TGF-β signaling in lung fibroblasts^34^. Consistent with these findings, we observed that pharmacological inhibition of DDR1 and DDR2 effectively inhibited TGF-β1 and subsequent Smad signaling. Here, our results suggested that DDRs are involved in the activation of TGF-β/Smad signaling and pharmacological inhibition of DDRs attenuated pancreatic fibrosis by inhibiting TGF-β/Smad signaling (Fig. 8). The limitation of our study is that due to unavailability of selective inhibitors, we have chosen FDA approved clinically available IMT as a model inhibitor of DDR1/2 receptors, which also processes inhibitory effects on other tyrosine kinases. Nevertheless, the use of highly selective agents against DDR1 and 2 receptors along with gene silence and knockout models may provide better molecular understanding on these novel targets to explore for chronic pancreatitis therapy.

## Conclusion

Taken together, the data from this study strongly provides evidences that activation of DDR1 and DDR2 are predominantly implicated in the progression of CP and associated fibrosis. Further, upregulation of DDR1 and DDR2 is involved in the enhancement of pancreatic injury, PSCs activation and TGF-β/Smad signaling. In addition, pharmacological inhibition of DDRs provided promising protective effects against CP and associated fibrosis. In the light of our results, it was suggested that DDRs play a significant role in CP progression and targeting of DDRs could become a potential target for halting the progression of CP.

## Supplementary Information

Supplementary Information.

## Abbreviations

CP: Chronic pancreatitis
CTGF: Connective tissue growth factor
DDRs: Discoidin domain receptors
ECM: Extracellular matrix
IMT: Imatinib
NF-κB: Nuclear factor kappa B
PSCs: Pancreatic stellate cells
PDGF: Platelet-derived growth factor
α-SMA: α-Smooth muscle actin
TGF-β: Transforming growth factor-β
