# Supplementary material for: Inhibition of discoidin domain receptors by imatinib prevented pancreatic fibrosis demonstrated in experimental chronic pancreatitis model
Source: Sci Rep. 2021 Jun 18;11:12894. doi: 10.1038/s41598-021-92461-z (PMC8213731; doi:10.1038/s41598-021-92461-z)
Supplement: Supplementary file 1 — Supplementary Information. [file 41598_2021_92461_MOESM1_ESM.docx]

**Inhibition of discoidin domain receptors by imatinib prevented pancreatic fibrosis demonstrated in experimental chronic pancreatitis model**

**Sapana Bansod^1, 2^, Mohd Aslam Saifi^1^, Chandraiah Godugu^1^***

^1^Department of Regulatory Toxicology, National Institute of Pharmaceutical Education and Research (NIPER), Balanagar, Hyderabad, Telangana, India

^2^Division of Oncology, Department of Internal Medicine, Washington University of School of Medicine, St Louis, Missouri, USA

**Running Head:** DDRs receptors: a promising target for chronic pancreatitis

***Correspondence:**

Dr. Chandraiah Godugu, Assistant Professor,

Department of Regulatory Toxicology,

National Institute of Pharmaceutical Education and Research (NIPER),

Balanagar, Hyderabad,

Telangana State, India-500037

Telephone: 040-23073741 Fax: 040-23073751

**Materials and Methods**

**Statement**

Animals were kept in well-controlled housing facilities at temperature (25 ± 2°C) and 12/12 hr light/dark cycle with free access to water as ad libitum and pellet food. The Animal experiments were designed, conducted and reported as per the ARRIVE guidelines [[18](#_ENREF_18)]. Specifically, all the animal experiments were performed according to the Committee for the Purpose of Control and Supervision of Experiments on Animals (CPCSEA), which is the approval body for animal experimentation in India. The CPCSEA certified Institutional Animal Ethical Committee (IAEC) of National Institute of Pharmaceutical Education and Research (NIPER)-Hyderabad, has reviewed the animal protocol and approved it (IAEC Protocol Approval No.: NIP/01/2019/RT/365).

Male Swiss albino mice (age: 6–8 weeks, body weight 25-30 g) were purchased from Palamur Biosciences Pvt. Ltd, Mahabubnagar, India. Mice were randomly divided into 6 groups (7 mice per group).

**Supplementary uncropped original western blots**

**Supplementary Figure S1**

**DDR1 (Fig 2A)**

**Actin (Fig 2A)**

**pDDR1 (Fig 2A)**

**125 kDa**

**125 kDa**

**43 kDa**

**110 kDa**

**116 kDa**

**DDR2 (Fig 2A)**

**Collagen1a (Fig 2A)**

**Figure S1:** Original western blots for images shown in Figure 2 of the main manuscript. The uncropped images are shown here.

**Supplementary Figure S2**

**Actin (Fig 3A)**

**DDR1 (Fig 3A)**

**DDR2 (Fig 3A)**

**Collagen1a (Fig 3A)**

**Collagen3a (Fig 3A)**

**110 kDa**

**100-110 kDa**

**125 kDa**

**125 kDa**

**pDDR1 (Fig 3A)**

**116 kDa**

**43 kDa**

**Figure S2:** Original western blots for images shown in Figure 3 of the main manuscript. The uncropped images are shown here.

**Supplementary Figure S3**

**NF-κB (Fig 5D)**


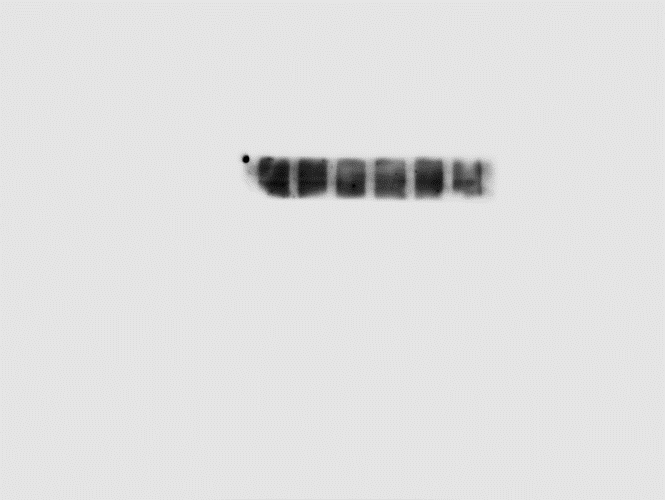

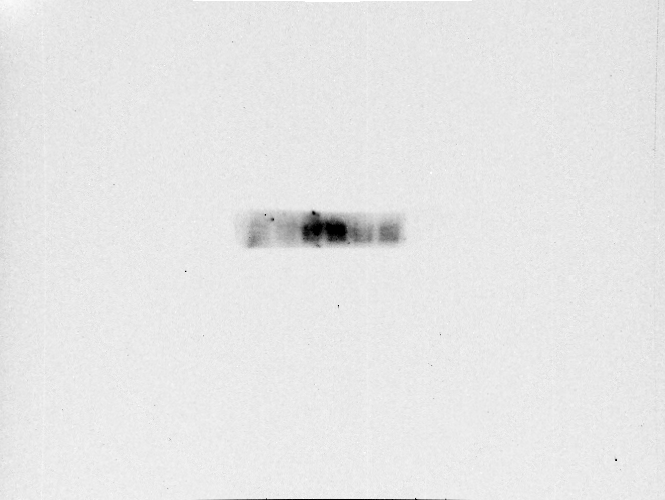

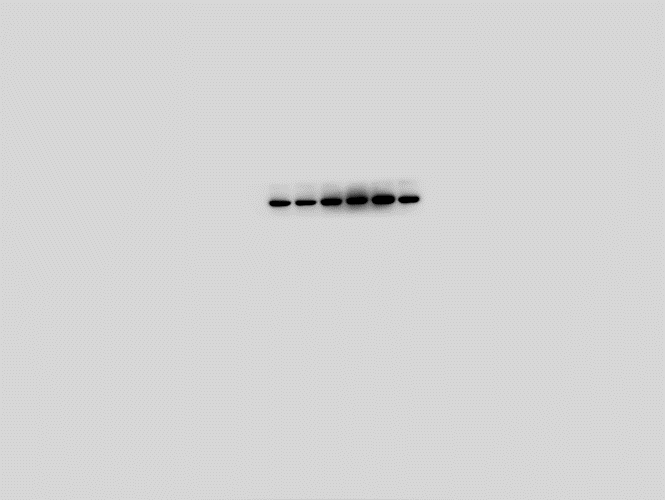


**pNF-κB (Fig 5D)**

**Actin (Fig 5D)**

**65 kDa**

**43 kDa**

**65 kDa**

**Figure S3:** Original western blots for images shown in Figure 5 of the main manuscript. The uncropped images are shown here.

**Supplementary Figure S4**

**Actin (Fig 6C)**

**α-SMA (Fig 6C)**

**42 kDa**

**43 kDa**

**Figure S4:** Original western blots for images shown in Figure 6 of the main manuscript. The uncropped images are shown here.

**Supplementary Figure S5**

**Actin** (**Fig 7A)**

**pSmad2/3** (**Fig 7A)**

**Smad2/3** (**Fig 7A)**

**38 kDa**

**43 kDa**

**15-25 kDa**

**52-60 kDa**

**52-60 kDa**

**CTGF** (**Fig 7A)**

**TGF-β1** (**Fig 7A)**

**Figure S5:** Original western blots for images shown in Figure 7 of the main manuscript. The uncropped images are shown here.
